# The impact of closing schools on working from home during the COVID-19 pandemic: evidence using panel data from Japan

**DOI:** 10.1007/s11150-020-09536-5

**Published:** 2021-01-11

**Authors:** Eiji Yamamura, Yoshiro Tsustsui

**Affiliations:** 1 Department of Economics, Seinan Gakuin University, 6-2-92 Nishijin, Sawara-ku, Fukuoka, 814-8511 Japan; 2 Department of Sociology, Kyoto Bunkyo University, Kyoto, Japan

**Keywords:** COVID-19, Gender difference, School closure, Primary school, Remote work, D13, J12, J13, J16, I28

## Abstract

COVID-19 has led to the closure of various schools in Japan to cope with the pandemic. This study explores how school closure influences parents’ work style based on short panel data for the period of school closure from mid-March to mid-April 2020. Specifically, we analyze how the presence of their children influences parents’ work at home and examine how the effect differs by the parent’s gender. After controlling for various factors, we find that in cases where parents are full-time employees and the children are: (1) in primary school, mothers are more likely to work remotely, while fathers are less likely to do so and (2) in junior high school, the parents’ work styles are hardly affected. This shows that mothers shoulder the burden of working remotely and caring for small children at home, while fathers tend to work in the office and spend less time with their childcare at home. Inevitably, COVID-19 has increased the inequality in the burden of child care.

## Introduction

How and to what degree does COVID-19 affect households? Is there a gender difference in the effects of COVID-19 on work–life balance? These questions are critical as the COVID-19 pandemic spreads worldwide in 2020. Unexpected shocks, such as a great recession, affect modern life and thus, time allocation (e.g., Aguiar et al. 2013; Gorsuch 2016; Pabilonia 2017). The government has required people to work from home to contain the spread of COVID-19, which inevitably leads to recession. The COVID-19 pandemic has drastically changed working styles and time use in various countries. A lockdown to cope with COVID-19 has increased the percentage of people who stay at home by 8% across US counties (Brzezinski et al. 2020). However, people with lower-paying jobs are less able to work from home in the United Kingdom (Costa-Dias et al. 2020). In contrast to other types of recessions, the recession caused by COVID-19 has a greater impact on sectors with high female employment shares (Alon et al. 2020).

COVID-19 has led to the closure of schools in various countries (Baldwin and Mauro 2020). Above all, the closure of primary schools has necessitated childcare at home, which inevitably has increased parents’ burden of childcare.^1^ Particularly, in a two-income household, a problem arises regarding who should care for the child at home. Married mother whose child delayed school entry increases labor supply for married women (Barua 2014). In Italy, most of the additional housework and childcare associated to COVID-19 falls on women while childcare activities are more equally shared within the couple than housework activities under COVID-19 pandemic (Del Boca et al. 2020).^2^ Does this hold in different cultural and social condition?

Primary and junior high schools were closed throughout Japan after Mach 2. However, there is a difference between Japan and other countries that implemented the policy of school closure. Under the lockdown adopted in various countries such as the United States, United Kingdom, France, and Italy, neither the firm’s manager nor the employees themselves can decide whether to go to the workplace. Meanwhile, the Japanese government has declared a state of emergency, but did not mandate workers to go to work. Those who choose to go their workplace are not penalized. Japanese workers can decide whether to go to the workplace or work from home to care for their child. Based on individual-level data collected directly after the closure of school in Japan, this study examines whether in a household with a child in primary school, the husband and/or wife work from home.

Existing research has evaluated the effect of school closure to cope with outbreaks of a number of viral diseases (e.g., Cauchemez et al. 2008, 2014; Adda 2016). Recent studies have considered the differences in COVID-19’s effects between genders (Adams 2020; Alon et al. 2020). Other works have analyzed home-stay behavior (Doganoglu and Ozdenoren 2020; Engle et al. 2020; Yamamura and Tsutsui 2020) and remote work (Hatayama et al. 2020) under COVID-19. However, other than Sevilla and Smith (2020), not many studies have investigated how school closure affects parents’ work from home and causes gender differences in work styles during the pandemic. This study contributes by analyzing the said topic in Japan, where workers can choose their working style even in an emergency situation.

The main findings are that in a household, males with children aged under 12 are less likely to work from home than other males, whereas females with children aged < 12 years are more likely to work from home than other females. However, having children in primary school does not significantly influence the work styles of parents in three-generation households. That is, grandparents informally provide childcare, which has reduced gender differences in parents’ work styles during the COVID-19 pandemic.^3^

The remainder of this article is organized as follows. Section 2 presents an overview of the situation in Japan and explains the surveys’ design. Section 3 describes the empirical method. Section 4 presents and interprets the estimated results. The final section provides some reflections and conclusions.

## Design of surveys and data

**Fig. 1:**
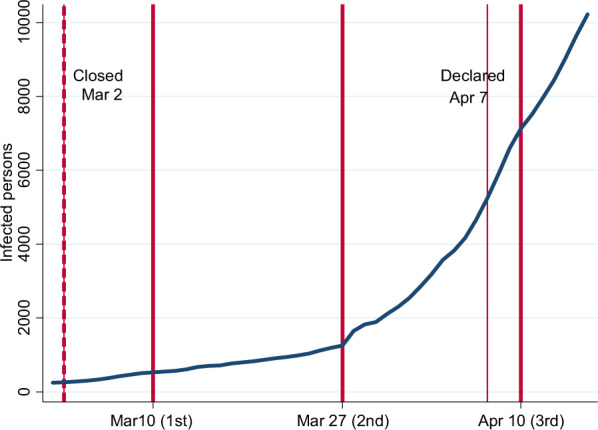
Timing of closing school and surveys

Figure 1 shows the changes in the total number of people infected with COVID-19 from Mach 1 to April 20. On February 27, the Japanese government requested schools to close beginning in March. The closure of various schools—primary, junior high, and high school—started on March 2 when the number of people infected with COVID-19 was only about 250. This number increased modestly and reached 1000 on March 24. Moreover, the plan to hold the 2020 Summer Olympics in Tokyo attracted criticism from citizens and other countries. Inevitably, on March 24, the Japanese government decided to postpone the Olympics for 1 year.

In April, the number of infected people increased drastically. In response, the government declared a state of emergency on April 7. Similar to other countries (Baldwin and Mauro 2020), art museums and amusement parks were closed, and professional sports games were canceled. To slow down the spread of COVID-19, people were requested to avoid persons-to-person contact and gathering together and were encouraged to stay at home. However, even in cases where people did not follow the request, these people were neither punished nor penalized under the state of emergency, which differed from the practice of the “lockdown” in the United States, the United Kingdom, Italy, France, and Spain. Therefore, Japanese citizens could actually behave based on their free will, although moral and informal social norms, to a certain extent, deterred them from practicing undesirable behavior (Yamamura 2009).

### Design of surveys

In response to the COVID-19 pandemic, in February 2020, we planned to conduct surveys to collect data for exploring the impact of COVID-19 on individual behaviors and households. INTAGE, a research company, has extensive experience in academic research. Because of its esteemed reputation and reliability, we commissioned INTAGE to conduct a survey through the Internet. The sampling method was designed to gather a representative sample of the Japanese population with regard to gender, age, educational background, and location of residence. Our survey selected Japanese citizens aged 16–79 throughout Japan. As illustrated in Fig. 1, the surveys were conducted three times to pursue the same individuals from March to April.

The first wave was conducted on March 13–16. We gathered 4359 observations, and the response rate was 54.7%. The second and third waves were conducted on March 27–30 and April 10–13. The response rates reached 80.2% (second wave) and 92.2% (third wave). The total number of observations was 11,867.

### Data

The components of the research sample are shown in Table 1. In Appendix, we provide the information showing that the data used for this analysis is similar to the data based nationally-representative surveys. This study examines how workers have changed their way of working during the COVID-19 pandemic. Hence, we limit the sample to workers. Further, the effect of having a child in primary school is considered. We assume that respondents who have children in primary school are aged below 50 years. To compare people with similar characteristics, we further limit the sample to respondents aged below 50. In the alternative sample, respondents are aged below 40. Part-time workers are more likely to lose their jobs than full-time workers, especially in emergency situations. Salaried and formal workers have jobs that are more amenable to working from home than the average workers (Hatayama et al. 2020).　To scrutinize the effect of having children who attend school, it is important to compare people that have similar jobs. Hence, we further limited the sample to full-time workers because they are less likely to lose their jobs. The main sample includes respondents who are workers aged below 50, with 2436 and 2009 observations for the male and female sub-samples, respectively. In each sample, observations with children in primary school total 626 and 528. Thus, ~25 % of the respondents have children in primary school. We used various sub-samples to check the robustness of the estimation results of the main sample.

**Table 1:** Construction of research sample: number in sample

Description	Male	Female
Original sample	5880 (777)	5987 (997)
Sample with variables used in estimations	5211 (719)	5326 (909)
Respondent is worker	4377 (707)	3612 (572)
Ages ≤ 50^a^	2436 (626)	2009 (528)
Ages ≤ 40^b^	1391 (341)	1205 (368)
Respondent is full-time employed worker	1617 (245)	1627 (268)
Ages ≤ 50^c^	794 (213)	783 (249)
Ages ≤ 40^d^	465 (121)	471 (155)

Number in parentheses show observations of respondents with primary school pupil. Various types of workers were classified as “worker” which include not only full-time employed workers but also self-employment, part-time worker, a temporary employee, professional worker such as lawyers and tax accountant

**Fig. 2:**
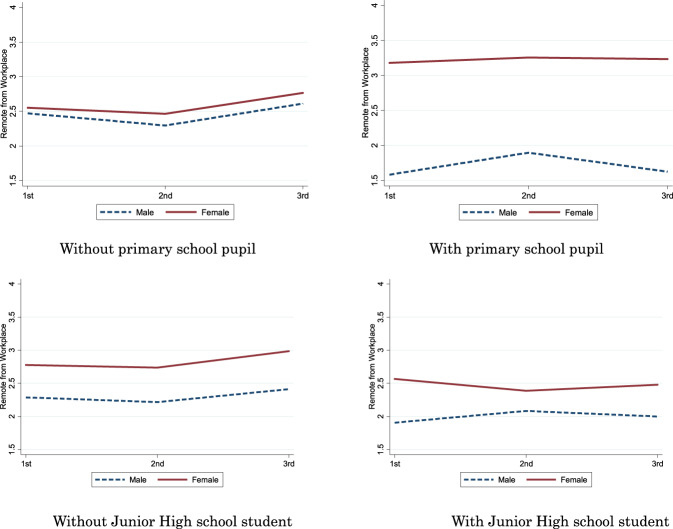
Change of “Work from home” and its gender difference according to having primary school pupil. Sample of ages ≤ 50 and having full-time employed workers

**Fig. 3:**
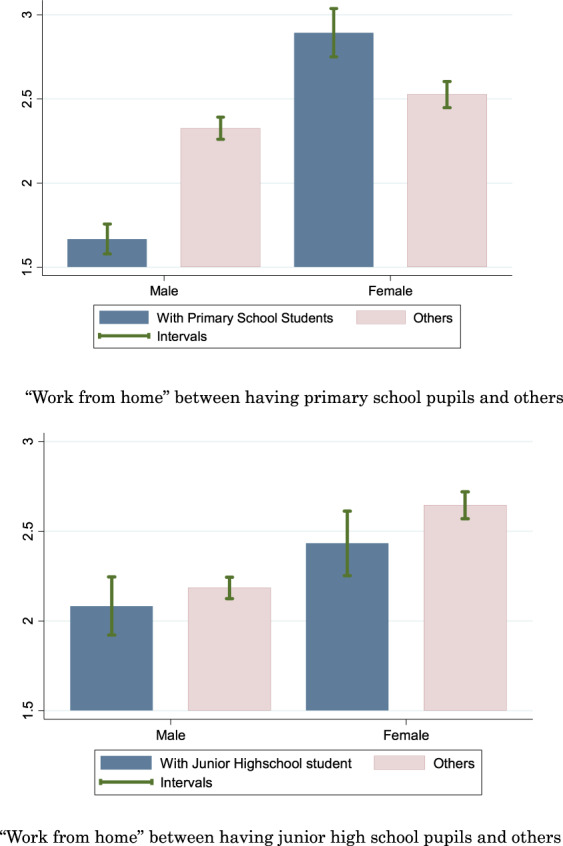
Comparison of “Work from home” between having school students and others. Sample is group of workers under 50 ages

**Fig. 4:**
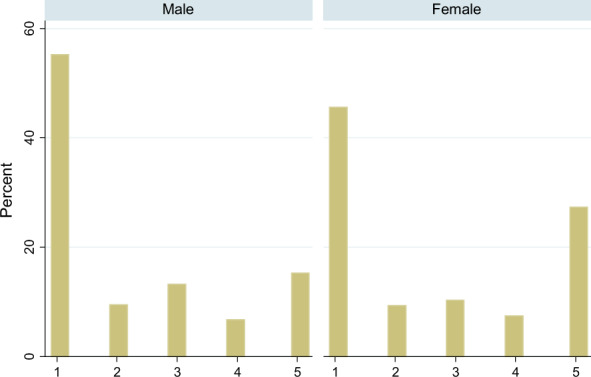
Histogram of degree of “Work from home” by comparing genders. Sample is group of workers under 50 ages

^a^Sample was used for estimations reported in Tables 2−4, Figs. 2–4

^b^Sample was used for estimations reported in Table 4

^c, d^Sample was used for estimations reported in Tables 5, 7 and 8

The descriptions of the variables used in this study are shown in Table 2. The survey questionnaire contained basic questions about demographics such as age, gender, educational background, household income, and job status, and about having children in primary school or junior high school. The job status has ten categories.^4^ Full-time workers are classified into three categories.^5^ Workers are further classified into seven categories.^6^ We assumed that the job status did not change because the three waves were conducted within a month. However, it is possible that some full-time workers lost their jobs during the recession caused by the COVID-19 pandemic. Therefore, in addition to the estimation results using a combined sample of waves 1–3, we also report the results using the sample of Wave 1, in which we asked respondents their job status.^7^ Therefore, the respondents’ job statuses are correct in the Wave 1 sample, even if they lost their jobs thereafter. *Primary School*, a dummy for having a child in primary school, is a key independent variable.

**Table 2:** Definition and basic statistics

	Definition	(1) Male	(2) Female
Work from home	“Within a week, to what degree have you achieved ‘not go to the workplace'? 1 (I have not achieved this behavior at all) to 5 (I have completely achieved this behavior).”	2.16	2.59
Primary school	Equals 1 if respondent’s child is primary school pupil, 0 otherwise	0.23	0.24
Junior high school	Equals 1 if respondent’s child is junior high school student, 0 otherwise	0.13	0.14
Schooling years	Respondent’s schooling years	14.5	14.2
Income	Respondent’s annual household income. (Million yen)	5.75	5.56
Ages	Respondent’s ages	34.9	34.2
Ages square	Square of respondent’s schooling years	1322	1274
Wave 2	Equals 1 if survey is second wave, 0 otherwise	0.29	0.26
Wave 3	Equals 1 if survey is third wave, 0 otherwise	0.35	0.41

Sample is limited to workers under 50 years old

In waves 1–3, respondents were asked the following questions:

“Within a week, to what degree have you practiced the behavior of ‘not going to the workplace’? Please answer based on a scale from 1 (I have not practiced this behavior at all) to 5 (I have completely practiced this behavior).”

We assumed that “not going to the workplace” meant “working from home” rather than “taking leave” because the Japanese Government had requested citizens to work from home.^8^ The answers to these questions served as proxies for the degree of “*Work from Home*.”

Figure 2 illustrates the changes in “*Work from Home*” as time passed and compares the difference between genders.

Figure 2 shows that female workers’ work-from-home behavior is almost the same as that of male workers if they do not have children in primary school. It is surprising that female workers’ work-from-home behavior is distinctly higher than that of male workers if they have children in primary school. Therefore, for parents with children in primary school, the mothers tend to work from home and the fathers tend to work in the workplace. As for workers with children in junior high school and others, there is no obvious difference observed. Taken together, Figs. 1 and 2 reveal that the work-from-home behavior remained stable from waves 1 to 3, although COVID-19 spread drastically and the state of emergency was declared during this period.

Turning to Fig. 3, the upper part indicates that males with children in primary school were less likely to work from home than other males. Meanwhile, females with children in primary school were more likely to work from home than other females. Therefore, the gender gap for Japanese citizens with children in primary school is very large, while the gender gap for those who do not have children in primary school is very small. The lower part demonstrates that there is no difference between citizens with children in junior high school and others. This is observed not only for males but also for females. The findings in Fig. 3 are consistent with the argument of Hatayama et al. (2020) that women tend to have jobs that are more amenable to working from home than the average worker. However, within the same gender, there is remarkable difference in the work-from-home behavior according to whether citizens have children in primary school, although having children in junior high school did not make difference. Overall, the observations in Fig. 3 suggest that parental care for children in primary school is more essential than that for children in junior high school during the COVID-19 pandemic, likely because primary school students are less mature.

Figure 4 demonstrates the distribution of work-from-home behavior. Around 30% of female and 15% of male respondents completely practiced “work from home” (i.e., those who responded “5” to the questionnaire). Meanwhile, around 45% of female and 55% of male respondents did not practice this behavior at all (i.e., those who responded “1”). The females’ work behavior is distributed between the two extremes, whereas that of the males’ is concentrated in “1.” That is, the male respondents mostly have not practiced the behavior at all.

Panel A of Table 3 compares the characteristics of respondents with children in primary school and others. As a whole, respondents with children in primary school are more educated, earn higher income, and are older than others. In Panel B, a similar tendency is observed, except for the educational background. We should take these differences into account when we examine the effect of having school-aged children. As explained in the next section, in addition to the OLS (Ordinary Least Square) model, we conduct a propensity score matching (PSM) estimation to compare respondents with similar characteristics.

**Table 3:** Mean difference test

Panel A: between respondents having primary school pupil and others			
	Primary school = 1 (1)	Primary school = 0 (2)	Difference (1) − (2)
Male sample			
Schooling years	14.7	14.4	0.24**
Income	7.00	5.38	1.61***
Ages	38.4	33.8	4.60***
Female sample			
Schooling years	14.1	14.2	−0.01
Income	6.17	5.38	0.79***
Ages	35.9	33.6	2.33***

Panel B: between respondents having junior high school student and others			
	Junior high school = 1 (1)	Junior high school = 0 (2)	Difference (1) − (2)
Male sample			
Schooling years	14.3	14.6	−0.30**
Income	7.05	5.56	1.49***
Ages	38.7	34.7	4.40***
Female sample			
Schooling years	13.5	14.3	−0.79***
Income	6.18	5.46	0.71***
Ages	38.4	33.4	4.96***

Sample is limited to workers under 50 years old

**, and *** denote statistical significance at the 5%, and 1% levels, respectively

## Methodology

### OLS model

We used a simple OLS regression model.^9^ The estimated function takes the following form:^10^

1 $$\begin{array}{l}Work\,from\,Home_{it} = \alpha _0 + \alpha _1Primary\,School_i + \alpha _2Junior\,High\,School_i\\ +\, \alpha _3Years\,of\,Schooling_i + \alpha _4Income_i + \alpha _5Age_i + \alpha _6Age\,Squares_i + \alpha _7Wave\,2_t\\ + \,\alpha _8Wave\,3t + u_{it},\end{array}$$

In this formula, *Work from Home*_*it*_ represents the dependent variable for individual *i* and wave *t*. *Primary School* is the key independent variable for exploring the effects of having children in primary school. To check for differences in the requirements of child care, *Junior High School*, a dummy for having a child in junior high school, is included in the estimations.^11^ The second (*Wave 2*) and third wave (*Wave 3*) dummies are included; their reference group is the first wave. These dummies capture the degree of change in the dependent variables compared with the first wave. The regression parameters are denoted as *α*. The error term is denoted by *u*. The data structure is a panel. However, we did not employ the fixed effects estimation because *Primary School* is constant and therefore, captured as fixed effects. Accordingly, the estimation results of *Primary School* cannot be obtained.^12^

We divided the sample into males and females to conduct the model for gender comparison. Furthermore, the situation in Japan drastically changed during the study period, as illustrated in Fig. 1. Therefore, we conducted the same estimations using a sub-sample of Wave 1 to focus on the situation before the rapid spread of COVID-19 and the declaration of the state of emergency. In addition, we can correctly identify the respondent’s job status in Wave 1.

### Propensity score matching estimation

We examined an alternative estimation, the PSM estimation. PSM estimates the average treatment effect (ATE) on the treated group to find a comparable observation in the untreated group (Abadie and Imbens 2016). To do this, we applied Eq. (2) to estimate the propensity score for each subject:

2 $$\begin{array}{l}{\mathrm{Prob}}\,\left( {Primary\,Student = 1} \right) = \\ L\left( {\beta _0 + \beta _1Age_i + \beta _2Years\,of\,Schooling_i + v_i} \right)\end{array}$$

Here, Prob and *L* stand for probability and the logistic function, respectively. The other variables were not included because they are not predetermined by *Primary Student*. As shown in Table 3, *Primary Student* is influenced by *Age* and Y*ears of Schooling*. However, the income level is not considered predetermined because having a child changes the work style, leading to a change in income. Accordingly, income was not included in Eq. (2). In the same framework, the probability of a *Junior High Student* was estimated. We estimated ATE by matching each subject to a single subject with the opposite treatment whose propensity score is the closest.

## Results and interpretation

### OLS estimation

Table 4 reports the results based on a sample of workers aged 50 and 40. We see from Table 4 that the coefficients of *Primary School* are negative and positive for males and females, respectively. Furthermore, the results are statistically significant at the 1% level. This suggests that in households with children in primary school, working mothers are more likely to work from home, while fathers are less likely to work from home. *Junior High School* does not show statistical significance in any column. This indicates that children in primary school influence their parents’ way of working, whereas children in junior high school do not. This reflects a difference in the necessity for childcare between children in primary school versus those in junior high school, who are considered to be more mature.

**Table 4:** Estimation results of the baseline model (dependent variable is “Work from home”): workers including part-time job

	Ages ≤ 50		Ages ≤ 40	
	(1) Male	(2) Female	(3) Male	(4) Female
Primary school	−0.33*** (0.07)	0.51*** (0.13)	−0.46*** (0.14)	0.68*** (0.15)
Junior high school	−0.14 (0.09)	−0.12 (0.13)	−0.07 (0.29)	−0.17 (0.31)
Schooling years	−0.02 (0.02)	−0.001 (0.02)	−0.02 (0.02)	0.02 (0.02)
Income	−0.10 (0.15)	0.25*** (0.09)	−0.03 (0.16)	0.46*** (0.13)
Ages	−0.19*** (0.02)	−0.15*** (0.02)	−0.41*** (0.09)	−0.27*** (0.09)
Ages square	0.002*** (0.0003)	0.002*** (0.0007)	0.006*** (0.001)	0.004** (0.002)
Wave 1	Reference		Reference	
Wave 2	0.04 (0.05)	0.04 (0.06)	0.05 (0.05)	0.16** (0.08)
Wave 3	0.52*** (0.09)	0.98*** (0.09)	0.55*** (0.11)	0.98*** (0.11)
R-squared	0.15	0.19	0.20	0.25
Observations	2436	2009	1391	1205

Numbers within parentheses are robust standard errors clustered by prefectures. In all columns, various control variables such as job dummies, current residential prefecture dummies, and a constant are included. However, these estimates are not reported

**, and *** denote statistical significance at the 5%, and 1% levels, respectively

Table 5 reports the results using a sample of full-time workers. Table 5 shows that *Primary School* has similar results to those in Table 4. That is, the sign of *Primary School* indicates a statistically significant difference between males and females. The absolute values of its coefficients are 0.41 for males and 0.85 for females, using sub-samples of participants aged below 50. This means that full-time male workers with children in primary school are 0.41 points less likely to work from home (on a five-point scale) compared with full-time male workers. Full-time female workers with children in primary school are 0.85 points more likely to work from home (on a five-point scale) compared with other full-time female workers.^13^ In short, the impact of *Primary School* on females is approximately two times larger than that on males, although the impact is opposite between the genders. One reason that fathers were more likely to work outside during school closure is that their kids stayed at home and play loudly. Also, an alternative explanation is that fathers worked outside because their wife stayed at home and helped them to do so during March-April. *Junior High School* shows a significant negative sign in columns (1) and (2), but this becomes insignificant in columns (3) and (4). That is, the effects of *Junior High School* are not robust.

**Table 5:** Estimation results (dependent variable is “Work from home”): full-time employed workers

	Ages ≤ 50		Ages ≤ 40	
	(1) Male	(2) Female	(3) Male	(4) Female
Primary school	−0.41** (0.15)	0.85*** (0.20)	−0.50** (0.20)	1.32*** (0.25)
Junior high school	−0.62*** (0.21)	−0.63* (0.37)	0.43 (0.47)	−0.49 (0.58)
Schooling years	−0.04 (0.04)	0.05 (0.05)	−0.11** (0.05)	−0.01 (0.07)
Income	−0.18 (0.24)	−0.28 (0.29)	0.07 (0.27)	0.04 (0.32)
Ages	−0.17** (0.07)	−0.35*** (0.10)	−0.15 (0.17)	0.01 (024)
Ages square	0.002** (0.001)	0.005*** (0.001)	0.002 (0.003)	−0.001 (0.004)
Wave 1	Reference		Reference	
Wave 2	0.01 (0.08)	−0.03 (0.08)	−0.06 (0.11)	0.04 (0.11)
Wave 3	0.23* (0.12)	0.28** (0.13)	0.19 (0.12)	0.33** (0.14)
R-squared	0.18	0.18	0.14	0.28
Observations	794	783	465	471

Numbers within parentheses are robust standard errors clustered by prefectures. In all columns, various control variables such as job dummies, current residential prefecture dummies, and a constant are included. However, these estimates are not reported

*, **, and *** denote statistical significance at the 10%, 5%, and 1% levels, respectively

In Table 5, as for the control variables that show statistical significance, in columns (1) and (2), *Age* and *Age Square* show negative and positive signs, respectively. This means that the older the workers are, the less likely they are to work from home, although the effect is diminished as the workers get older. In our interpretation, older workers are more likely to hold a position of responsibility; thus, they are required to work in the office to deal with complicated and difficult problems, even during the COVID-19 pandemic. We observe a significant positive sign for *Wave 3*. As shown in Fig. 1, *Wave 3* was conducted after the state of emergency was declared. Naturally, full-time workers were more likely to adhere to the request of working from home at the time of *Wave 3*. The absolute values of the coefficients are 0.23 and 0.28 for males and females, respectively. The absolute values of the coefficients of *Primary School* and *Wave 3* jointly indicate that for a female having a child in primary school increases of *Work from Home*, which is roughly three times more than increase of *Work from Home* from the first wave to the third wave.

Drastic changes in the situation possibly increased the number of unemployed workers. As shown earlier, the question about *Work from Home* relates to whether respondents do not go to the workplace. There is a possibility that people who lost their jobs after *Wave 2* and *Wave 3* are included in those who work from home. To reduce the measurement error, we limit the sample to Wave 1 since at that time we asked respondents their work status. The results of the Wave 1 sub-sample are shown in Panel A of Table 6, although we only report the results of key variables. With respect to the female sample, *Primary School* continues to show a positive sign with statistical significance at the 1% level. For the male sample, *Primary School* becomes insignificant in column (3). Panel B reports the results based on Waves 1 and 2 sub-samples, which are similar to those in Panel A. The effects of *Primary School* are robust to alternative sub-samples.

**Table 6:** Estimation results (dependent variable is “Work from home”): robustness check for key variables using various sub-samples

Sample	Ages ≤ 50		Ages ≤ 40	
	(1) Male	(2) Female	(3) Male	(4) Female
Panel A: sub-sample of wave1				
Primary school	−0.52** (0.20)	0.89*** (0.22)	−0.54 (0.34)	1.71*** (0.32)
Junior high school	−0.63** (0.27)	−0.53 (0.43)	0.80 (1.53)	−0.74 (0.84)
R-squared	0.23	0.21	0.34	0.41
Observations	267	264	147	153

Panel B: sub-sample of waves 1 and 2				
Primary school	−0.37* (0.19)	0.97*** (0.21)	−0.42 (0.29)	1.62*** (0.27)
Junior high school	−0.72*** (0.23)	−0.62 (0.41)	0.02 (0.88)	0.63 (0.78)
R-squared	0.20	0.20	0.33	0.38
Observations	487	479	275	278

Numbers within parentheses are robust standard errors clustered by prefectures. In all columns, various control variables such as job dummies, current residential prefecture dummies, and a constant are included. However, these estimates are not reported

*, **, and *** denote statistical significance at the 10%, 5%, and 1% levels, respectively

Overall, the combined results in Tables 4–6 reveal that female workers are likely to work from home if they have a child in primary school, while the opposite effect is observed for male workers. According to existing works (Aguiar et al. 2013; Gimenez-Nadal and Molina 2014), in the previous periods of economic recession, males were observed to allocate more time to childcare, which is contradictory to our findings in this recession caused by the COVID-19 pandemic.

It is widely observed that mothers with small children have a better chance to work if grandparents live with them and care for the children (e.g., Ho 2015; Bratti et al. 2018; Takaku 2019). If this holds true, *Primary School* would not influence *Work from Home* in a household with three-generations. To test this assumption, we conducted estimations using a sub-sample of households with three-generations.^14^ In Table 7, the statistical significance of *Primary School* disappears in all columns. This clearly shows that the closure of school has no impact on the working styles of working fathers and mothers. This is consistent with the argument of existing studies.

**Table 7:** Estimation results (dependent variable is “Work from home”): examining effect of grand-parents

Three-generation households of full-time employed workers				
Sample	All ages		Ages ≤ 50	
	(1) Male	(2) Female	(3) Male	(4) Female
Primary school	0.57 (0.69)	−0.17 (0.35)	−0.32 (0.70)	0.61 (0.90)
Junior high school	−0.39 (0.55)	0.08 (0.52)	0.35 (0.87)	0.49 (0.87)
R-squared	0.34	0.26	0.44	0.38
Observations	493	562	174	173

Numbers within parentheses are robust standard errors clustered by prefectures. In all columns, various control variables such as job dummies, current residential prefecture dummies, and a constant are included. However, these estimates are not reported

### Propensity score matching estimation

For the PSM analysis to be valid, we checked whether the estimated propensity score adequately balances the characteristics of the treated and untreated groups.^15^ In our case, the covariates to estimate the propensity score are *Age* and Y*ears of Schooling*. Therefore, we checked whether these two variables become closer by matching between those that have children in primary school (junior high school) and others. Specifically, we calculated the covariates’ standardized difference and the variance ratio between them. The balancing condition requires that (1) the standardized difference for the matched data is closer to 0 than for the raw data, and (2) the variance ratio for the matched data is closer to 1 than for the raw data.

In Table 8, the results of the covariate balance check are presented. In Panel A, checking *Primary School*, the standardized difference in *Age* decreases from 0.29 for the raw sample to −0.06 for the matched sample,^16^ while the variance ratio shown in parentheses increases from 0.49 to 0.94. The results for *Years of Schooling* also show that the matched samples are well-balanced. The results of the covariate check for *Junior High School* are presented in Panel B. Similar to Panel A, both *Age* and *Years of Schooling* are balanced. These results suggest that the propensity score matching method is valid.

**Table 8:** Covariate balance check: propensity score matching estimation (sample: Age ≤ 50, Full-time employed workers)

	Male		Female	
	Raw	Matched	Raw	Matched
Panel A: primary school				
Ages	0.29 (0.49)	−0.06 (0.94)	0.15 (0.52)	−0.06 (0.87)
Schooling years	0.17 (0.87)	0.09 (1.11)	−0.13 (0.98)	−0.06 (1.03)
Panel B: junior high school				
Ages	0.48 (1.14)	−0.004 (1.04)	0.58 (1.17)	−0.02 (1.11)
Schooling years	−0.05 (0.69)	−0.02 (0.77)	−0.42 (0.70)	−0.03 (1.36)

Values without parentheses are the standardized difference between those having primary school pupils (Junior high school student) and others. Values within parentheses are the variance ratios between them

Table 9 presents the average treatment effect (ATE) of having children in primacy school on *Work from Home*. We specify the maximum distance (caliper) to be 0.03, for which two observations are potential neighbors. With respect to the upper part, to show the results of *Primary School*, with the exception of column (1), all observations have propensity score matches within the caliper of 0.03. *Primary School* shows a negative (positive) sign for the male (female) sample. We observe statistical significance at the 1 % level in all columns. These results are consistent with Tables 4–6. Furthermore, for columns (1) and (2), the absolute values of the coefficients are 0.40 and 0.84 for males and females, respectively. These values are almost equivalent to the values reported in columns (1) and (2) of Table 5. These results indicate that the estimation results of *Primary School* are very robust and reliable. In the lower part of the table, *Junior High School* shows statistical significance only for the male sample. Further, its coefficient’s sign varies according to the sub-sample. Hence, the effects of *Junior High School* are not clear.

**Table 9:** Propensity Score matching model (dependent variable is “Work from home”)

	Age ≤ 50 Full-time employed workers		Age ≤ 40 Full-time employed workers	
	(1) Male	(2) Female	(3) Male	(4) Female
Primary school	−0.40*** (0.13)	0.84*** (0.18)	−0.62*** (0.19)	1.19*** (0.20)
Observations	779	777	121	471
Treated observation	213	246	448	155
Excluded observations	15	0	0	0
Junior high school	−0.33* (0.18)	−0.32 (0.23)	0.79** (0.39)	−0.58 (0.41)
Observations	794	780	454	463
Treated observation	79	113	19	32
Excluded observations	0	3	11	8

Average treatment effect (ATE) of having primary school pupil (junior high school student) on remote work. The Logit model is used for calculating the propensity score. Abadie–Imbens robust standard errors are shown in parentheses. “Excluded observations” shows number of observations are excluded because these observations have no propensity-score matches within caliper (0.3)

The symbols *, **, and *** denote statistical significance at the 10%, 5%, and 1% levels, respectively

Overall, the results of *Primary School* are robust to alternative specifications. What we found in this study partly reflects the fact that females’ jobs are more amenable to working from home (Hatayama et al. 2020). In the post-COVID-19 period, it is plausible that females are more able to work from home, which improves women’s social status. In the workplace, managers instruct working mothers with small children to work from home in consideration of their situation. Her husband might welcome that she works from home because he can focus on his work in the workplace. The spousal gap has changed the allocation of housework between husband and wife (Yamamura and Tsutsui 2021). The gender gap is very large in Japan (World Economic Forum 2020). Accordingly, in the case of female workers with small children, women work not only as salaried workers, but also as caregivers for their children. Yamamura and Tsutsui (2020) found that people’s mental condition worsened as the COVID-19 pandemic diffused. Within a household, the gender gap in the burden of work may have a negative influence on the mental condition of the mother and her child.^17^ An increase in stress may lead a mother to abuse her child, which has a negative impact on child growth. Therefore, it is critical to consider how to maintain work–life balance and to reduce the gender gap between husband and wife for designing the post-COVID-19 society.

## Conclusion

The policy of school closure has been adopted in various countries to cope with the COVID-19 pandemic. During the period after the declaration of school closure in Japan, we collected data to explore whether school closure leads full-time workers with school-aged children to work from home. Further, we investigated whether the effect of school closure differed between genders. We found that children in primary school lead working mothers to work from home. Meanwhile, fathers with primary-school-aged children are less likely to work from home than other male workers. This implies that the COVID-19 pandemic has increased the gender childcare gap and increased the burden of wives who work not only as remote workers but also as housewives.

Our findings are consistent with the argument of gender identity (Akerlof and Kranton 2000). By contrast, Sevilla and Smith (2020) analyzed gender allocation of childcare within couples with children aged < 12 years in the United Kingdom and found that the gender childcare gap has become smaller than before the COVID-19 pandemic.

Different findings from the United Kingdom and Japan can be interpreted in various ways. Japanese workers are more loosely controlled by the government than in the United Kingdom. Hence, male workers can work in the workplace, which explains the difference. From a different perspective, women’s social status in Japan is far lower than that of women in the United Kingdom (World Economic Forum 2020). Therefore, due to differences in bargaining power, working mothers are more likely to shoulder a larger burden. However, the increase in female burden generates stress, not only on herself but also on her child. From a long-term perspective, this has a detrimental effect on women’s mental health and their children’s growth. Thus, it is important for researchers to investigate the reason for the different impacts of COVID-19 on the gender gap in childcare between the United Kingdom and Japan. In future research, we should examine how the effects of COVID-19 differed between different cultural and economic settings by using more sophisticated approach such as the DID method or experiments.

## Figures and Tables

**Fig. 5:**
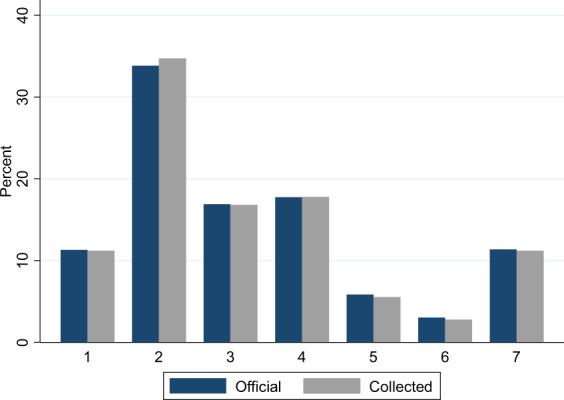
Comparing collected sample with the representative sample from government surveys in terms of residential regions. 1, 2, 3, 4, 5, 6, 7 in x-line mean Hokkaido-Tohoku, Knato, Chubu, Kansai, Chugoku, Shikoku, and Kyushu regions, respectively

**Fig. 6:**
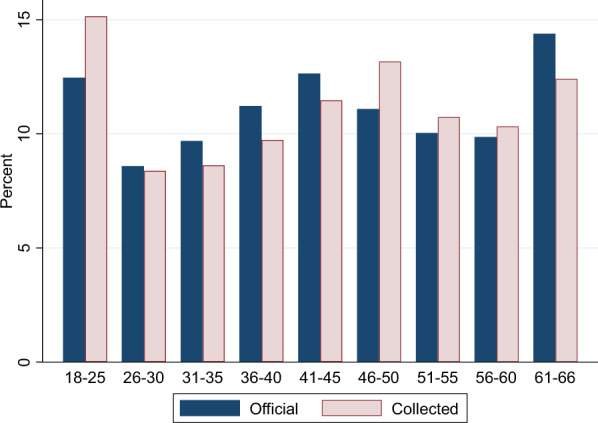
Comparing collected sample with the representative sample from government surveys in terms of ages. x-line indicates age group

**Fig. 7:**
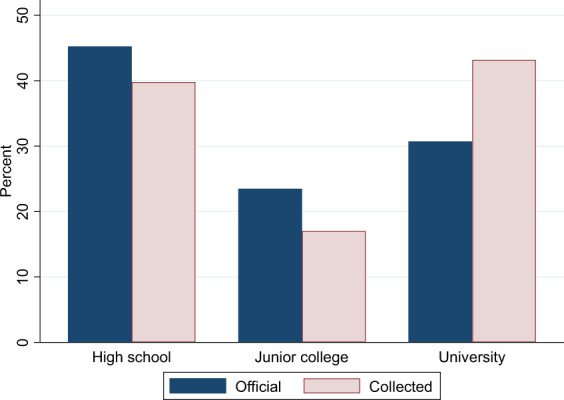
Comparing collected sample with the representative sample from government surveys in terms of educational background. Data is limited to workers. “High school” means schooling years being equal or lower than 12 years. “Junior college” means schooling years being equal to 14 years. “University” means schooling years being equal or higher than 16 years

## Appendix

Figs. 5–7

## Footnotes

1 Self-care after school leads to an increased risk of skipping school and use of alcohol and drugs (Aizer 2004). Economic recessions increase teenagers’ risky behaviors (Pabilonia 2017). A mother’s absence reduces the time a child spends in school (Pörtner 2016). It is important not to reduce child care during the recession caused by COVID-19.

2 Parental time with children is one of the major topics in the field of household economics (e.g., Gutiérrez-Domènech 2010; Aguiar et.al. 2013; Gimenez-Nadal and Molina 2014; Morrill and Pabilonia 2015; Gorsuch 2016; Romanm and Cortina 2016; Bauer and Sonchak 2017).

3 Several works deal with the relation between child care availability and maternal employment (e.g., van Gameren and Ooms 2009; Havnes and Mogstad 2011; Abe 2013; Asai et al. 2015; Brilli et al. 2016). Grandparents play a major role in enabling female labor participation (e.g., Ho 2015; Bratti et al. 2018; Takaku 2019).

4 These categories are (1) company employee, (2) manager in a company, (3) public employee, (4) freelancer, (5) independent professional, (6) temporary employee, (7) part-time worker, (8) student, (9) house worker, and (10) unemployed.

5 These categories are (1) company employee, (2) manager in a company, and (3) public employee.

6 Aside from full-time worker, the other categories are freelancer, independent professional, temporary employee, and part-time worker.

7 We did not ask about the job status in waves 2 and 3.

8 Some workers had to stop working completely during March. For example, most of the tasks of fulltime shop staff in boutique store were canceled if her store was closed. Probably because women are more likely to work at face-to-face jobs than men (Hatayama et al. 2020). So, there is possible interpretation that “not going to the workplace” meant just “not going to the workplace.”

9 *Work from Home* is an ordered, discrete variable. In this case, the ordered probit estimation is appropriate. However, its estimation results are similar to the results derived from the OLS model. Our argument does not change when using the ordered probit. However, the interpretation of the OLS results is simpler and easier to understand than that of the ordered probit model. Thus, OLS is used in this study. The results of the ordered probit model are available upon request from the corresponding author.

10 The difference in difference (DID) method is appropriate to examine impact of the school closure. However, unfortunately, we could not use the DID method because school closure has been conducted even in the first wave and throughout Japan. We would employ the DID method to re-examine the findings of the present study if we obtain the data which is appropriate for the DID method.

11 We only asked information about child’s school at March (in the first wave). We assume that children did not move up to the upper school during the studied period.

12 It is possible that primary school students entered junior high school in April if they were in sixth grade in March under the educational system of Japan. Similarly, junior high school students possibly entered high school in April if they were in third grade in March. However, we only asked respondents whether they have a child in primary school (junior high school) in Wave 1. Thus, we assume that *Primary School* and *Junior High School* are the same from Wave 1 to Wave 3.

13 As is shown in results presented in Tables 4 and 5, full-time workers may be more likely to work from home than part-time workers, at least among women. There might have been a group of working mothers who had no choice but to leave children alone at home or to quit their jobs because they did not have the option of working from home to stay with their children.

14 In the survey, we asked whether respondents live with people aged 70–79. In this study, a three-generation household is defined as a household where the respondent lives with people aged 70–79. There is possibility of measurement errors. Hence, careful attention is required to interpret the results of Table 7.

15 The covariates are balanced in the experimental data, as the treatment is independent of the covariates due to the design of the experiment. For data from the real world, the covariates must be balanced by matching observational data because the treatment is related to the covariates, which also affects the outcome of interest.

16 The standardized difference in *Age* for the row sample is its simple mean difference between groups.

17 There is an argument about the positive effect of women working from home. The lower rate of female deaths from COVID-19 is explained by the lower rate of women going to the workplace (Adams 2020). However, in the post-COVID-19 period, inevitably this benefit will disappear.
