# Correction to: The impact of closing schools on working from home during the COVID-19 pandemic: evidence using panel data from Japan

**DOI:** 10.1007/s11150-021-09548-9

**Published:** 2021-02-05

**Authors:** Eiji Yamamura, Yoshiro Tsustsui

**Affiliations:** 1 Department of Economics, Seinan Gakuin University, 6-2-92 Nishijin, Sawara-ku, Fukuoka, 814-8511 Japan; 2 Department of Sociology, Kyoto Bunkyo University, Kyoto, Japan

Correction to: *Review of Economics of the Household*

10.1007/s11150-020-09536-5

The article “The impact of closing schools on working from home during the COVID-19 pandemic: evidence using panel data from Japan”, written by Eiji Yamamura and Yoshiro Tsustsui, was originally published electronically on the publisher’s internet portal on 11 January 2021 without open access. With the author(s)’ decision to opt for Open Choice the copyright of the article changed on 18 January 2021 to © The Author(s) 2021 and the article is forthwith distributed under a Creative Commons Attribution 4.0 International License, which permits use, sharing, adaptation, distribution and reproduction in any medium or format, as long as you give appropriate credit to the original author(s) and the source, provide a link to the Creative Commons licence, and indicate if changes were made. The images or other third party material in this article are included in the article’s Creative Commons licence, unless indicated otherwise in a credit line to the material. If material is not included in the article’s Creative Commons licence and your intended use is not permitted by statutory regulation or exceeds the permitted use, you will need to obtain permission directly from the copyright holder. To view a copy of this licence, visit http://creativecommons.org/licenses/by/4.0.

